# Long-Term Risks of Benign and Malign Complications after Endoscopic Sphincterotomy in the Management of Benign Biliopancreatic Pathology: A Cohort Study

**DOI:** 10.5152/eurasianjmed.2024.23147

**Published:** 2024-02-01

**Authors:** Mercedes Ibáñez, Cristina López, Jeancarlos Trujillo, Juan Ramón Gómez, Carlos Vaquero Puerta, Juan Carlos Martin del Olmo

**Affiliations:** 1 Division of Digestive Diseases, Hospital of Medina del Campo, Valladolid, Spain; 2 Department of Microbiology, Hospital of Virgen de La Concha, Zamora, Spain; 3 Department of General Surgery, Hospital of Medina del Campo, Valladolid, Spain; 4 Department of Surgery, University of Valladolid, Valladolid, Spain

**Keywords:** Cholangitis, choledocholithiasis, complications, endoscopic retrograde cholangiopancreatography, pancreatitis

## Abstract

**Background:**

Endoscopic retrograde cholangiopancreatography (ERCP) is the preferred technique for treating pathologies of the bile duct. It has been suggested that this procedure, combined with sphincterotomy, may influence the subsequent development of long-term complications. The main objective of this study was to determine the long-term complications of biliopancreatic disease after ERCP and their potential association with the development of biliopancreatic neoplasms.

**Methods:**

This retrospective cohort study included 576 patients who underwent ERCP (referred to as index ERCP) with sphincterotomy for benign biliary disease, with a minimum follow-up period of more than 2 years.

**Results:**

The incidence of long-term benign and neoplastic pathologies after ERCP was analyzed. The most common findings were recurrence of choledocholithiasis in 70 patients (12.1%), cholangitis “sine materia” in 27 patients (4.7%), and acute pancreatitis in 8 patients (1.4%). Eight patients (1.4%) developed hepatobiliopancreatic neoplasms, including 4 cases of pancreatic neoplasms (0.7%), 1 cholangiocarcinoma (0.2%), 1 ampulloma (0.2%), and 2 intrahepatic neoplasms (0.3%). Multivariate analysis revealed that factors such as age over 50 years, previous biliary surgery, diversion of the bile duct (BD) to the digestive tract, dilation of the BD, stent placement, biopsy, and cholecystectomy were factors associated with an increased risk of long-term benign complications.

**Conclusion:**

Endoscopic retrograde cholangiopancreatography with sphincterotomy is associated with an increased long-term risk of subsequent benign biliopancreatic disease. However, our data do not allow us to establish a direct relationship with the development of biliopancreatic neoplasms.

**Main Points**

- Our study provides evidence that performing an endoscopic retrograde cholangiopancreatography (ERCP) can lead to long-term benign complications in patients, with an incidence rate of 16.3%. Choledocholithiasis is identified as the most common long-term complication following ERCP with sphincterotomy.
- We observed that patients over 50 years of age at the time of ERCP, diversion of the bile duct to the digestive tract, and bile duct dilation above 8 mm were more likely to develop benign complications.
- Post-ERCP cholecystectomy is not associated with an overall decrease in biliopancreatic complications following ERCP.
- We were not able to find out the association between ERCP with sphincterotomy and the development of biliopancreatic tumors.

## Introduction

Endoscopic retrograde cholangiopancreatography (ERCP) is the preferred technique for diagnosing and treating pathologies of the bile duct.^1^ It is used for various indications such as biliary stenosis, choledocholithiasis, sphincter of Oddi dysfunction, management of complications of chronic pancreatitis, congenital malformations of the bile duct, and repair of bile duct damage resulting from surgical complications.^2^

Sphincteroplasty, sphincterotomy, and prosthesis placement are integral parts of the ERCP technique, offering benefits but also carrying risks and complications in both the short and long term.^3^

Prior to the development of ERCP, the diagnosis of benign bile duct pathology was made intraoperatively through direct cholangiography.^4^ This procedure was associated with significant morbidity and had a low diagnostic yield. Surgical intervention, typically choledochotomy with T-tube or biliary-enteric anastomosis (choledochoduodenostomy or choledochojejunostomy), was commonly performed for treating this pathology. However, these procedures were associated with considerable morbidity and mortality.^5,6^

Initially, the application of ERCP was limited to patients who were not suitable candidates for surgery due to advanced age or underlying diseases, mainly due to concerns about potential complications. However, as more evidence emerged regarding the reliability and efficacy of the procedure, the range of indications gradually expanded.^7^ In the context of cholelithiasis and concomitant choledocholithiasis, ERCP started being recommended before or after surgery to avoid open surgery and complications associated with bilioenteric anastomosis.

Nevertheless, studies have reported a higher incidence of long-term complications such as cholangitis and acute pancreatitis following ERCP with sphincterotomy.^8^ The possible explanation for these events appears to be related to duodenal reflux caused by the loss of sphincter of Oddi function.^9^ It is postulated that endoscopic sphincterotomy, particularly when combined with temporary stenting, can lead to reflux of intestinal contents into the bile duct, like what occurs during surgery. This reflux may cause changes in the epithelium of the bile duct, potentially leading to the development of long-term benign and malignant pathologies after ERCP.^10^

Among these complications, the most reported are recurrent choledocholithiasis, distal and papillary bile duct stenosis, ascending cholangitis “sine materia,” cholecystitis, and pancreatitis.^11^

The primary objective of this study was to investigate the long-term outcomes of biliopancreatic diseases following ERCP and explore any possible association with the development of benign or malignant biliopancreatic pathologies.

## Material and Methods

This retrospective cohort study followed the Strobe checklist and included all patients who underwent ERCP between 1997 and 2017. Data from 576 patients and 717 ERCP procedures for benign bile duct pathology were collected. As multiple procedures were performed in some cases, the initial procedure was identified as the index ERCP.

The study included patients who were aged 18 years or older, underwent ERCP with sphincterotomy, and had a follow-up period of more than 2 years. Patients with biliopancreatic malignancy and those lost to follow-up before 2 years were excluded.

Patient information, clinical variables, time of cholecystectomy, indications for ERCP, post-ERCP diagnoses, and complications occurring within 6 months after ERCP were retrospectively collected from the patients’ medical records. Finally, information on the end date of follow-up and the reason for ERCP were recorded.

In the univariate and multivariate analyses, several factors were found to be significantly associated (*P* < .05) with the development of complications.

### Statistical Analysis

The statistical analysis was conducted using the Statistical Package for Social Science Statistics software for Windows, version 25.0 (IBM SPSS Corp.; Armonk, NY, USA). Normally distributed data are presented as means (SDs), while variables that do not follow a normal distribution are presented as medians (ranges). The Student’s *t*-test was used to compare normally distributed continuous variables, while the Mann–Whitney *U*-test was used for variables that did not have a normal distribution. Categorical variables were compared using the chi-square test. Statistical significance was considered at *P* < .05. Receiver operating characteristic analysis was performed for each inflammatory score to determine optimal cutoff values, with CD≥III (Clavien-Dindo) complications serving as the reference variable. Logistic regression analysis was utilized to construct multivariate models.

### Ethical Concerns

This study involved the utilization of data extracted from clinical records. To ensure the appropriate handling of this information, strict confidentiality and anonymity measures were implemented in compliance with the Spanish Organic Law 15/1999, dated December 13, 1999, on Personal Data Protection. All methods employed in this study adhered to the guidelines and regulations outlined in the Declaration of Helsinki (1964, revised in 1983) regarding biomedical research involving human subjects, as well as the Spanish Royal Decree 1090/2015, dated December 4, which governs clinical trials with drugs, Research Ethics Committees with drugs, and the Spanish Registry of Clinical Studies.

Ethical approval for this study was obtained from the Clinical Trials and Ethics Committee of Valladolid University (PI 18-889, Date: 02/07/2018).

## Results

A total of 576 patients met the inclusion criteria for the study. The average age of the patients was 71 years (interquartile range: 64-81, SD: 14.43). Among the sample, there were 269 male patients (46.5%) and 309 female patients (53.5%).

The average follow-up duration for the patients was 131 months (interquartile range: 69-149, SD: 63.96) (Table 1).

Regarding comorbidity factors, the following were notable: diabetes in 137 patients (23.7%), arterial hypertension in 384 patients (60.2%), and obesity in 142 patients (24.6%). Cholecystectomy was performed in 440 patients (76.4%) (Table 1).

The most common indication for ERCP was jaundice or analytical cholestasis in 159 cases (27.6%) and the main diagnosis made after ERCP was choledocholithiasis in 262 cases (45.5%) (Table 2).

A total of 141 cases (24.5%) required subsequent procedures after the index ERCP. Out of these, 94 successive ERCPs were performed for the treatment of complications (66%), and 47 ERCPs were performed for BD revision or removal of the prosthesis (33.3%) (Table 2).

We did not find any patients who required lithotripsy to extract lithiasis from the bile duct.

A total of 94 patients experienced long-term benign post-ERCP complications after the index ERCP, accounting for 16.3% of the cases. Among those that developed at least 6 months after the index ERCP, the most common complication was the recurrence of choledocholithiasis or bile duct sludge, which affected 68 patients (11.8%), with 15 patients (2.6%) experiencing multiple recurrences with 2 or more episodes. The second most frequent complication was ascending cholangitis “sine materia,” which affected 27 patients (4.7%), with repeat episodes occurring in 8 of these patients (1.4%). Acute pancreatitis was the third most common complication, affecting 8 patients (1.4%), with recurrences observed in 2 patients (0.3%). The number of complications was 102 because some patients had more than 1 complication (Table 3).

The factors associated with long-term benign complications included age over 50 years, diagnosis of biliary fistula, diversion of the bile duct to the digestive tract, dilated bile duct, performing cholecystectomy prior to ERCP, stent placement, cytology, and repeat ERCP. However, there was no significant increase in complications in the group of patients who underwent more than 1 ERCP procedure (Table 4 and Table 5).

Regarding the biliopancreatic neoplastic pathology detected during follow-up, a total of 8 neoplastic processes (1.4%) were recorded more than 2 years after the index ERCP. These included 4 pancreatic neoplasms (0.7%), 1 cholangiocarcinoma (0.2%), 2 intrahepatic neoplasms (0.3%), and 1 ampulloma (0.2%).

Factors such as chronic liver disease, chronic pancreatitis, bile duct dilation, cytology, diversion of the bile duct to the digestive tract, and stent placement were associated with malign occurrence.

## Discussion

Endoscopic retrograde cholangiopancreatography is a commonly used technique for the treatment of benign bile duct pathologies, but its association with short- and long-term complications remains unclear.^12,13^ However, our study provides evidence that performing an ERCP can lead to long-term complications in patients, with an incidence rate of 16.3%, which is consistent with previously published studies.^8,9,11,12,13^

It is postulated that the use of endoscopic or surgical sphincterotomy during ERCP may increase the risk of complications by allowing contaminated duodenal material to ascend into the bile duct, causing epithelial damage. This hypothesis supports the observed increase in the incidence of complications over time.^10^ As a result, unnecessary ERCP procedures in young patients, especially for diagnostic purposes, should be limited.^14^

Choledocholithiasis is identified as the most common long-term complication following ERCP with sphincterotomy,^13,15^ which aligns with our findings of an 11.8% incidence rate (n = 68). Additionally, 2.6% of these patients (n = 15) experienced multiple recurrences of choledocholithiasis. This incidence rate is consistent with the range of 10%-14.8% reported by Kanamori^15^ and higher than the 5% rate reported by Langerth.^13^ Our study identifies previous biliary surgery, cytology sampling, and stent placement as risk factors for recurrence of choledocholithiasis, which were not addressed in previous studies.

These findings highlight the importance of careful consideration and evaluation of the indications for ERCP, particularly in young patients, to minimize the risk of complications. 

Among the long-term complications observed in our study, the second most frequent complication was cholangitis “sine materia,” which refers to cholangitis without associated choledocholithiasis. The overall incidence of this complication was 4.7% (n = 27), with a repeated incidence of 1.4% (n = 8), which aligns with rates reported in the previous literature.^13,15^

The incidence of post-ERCP acute pancreatitis is estimated to be between 3% and 10% in general.^3,16^ However, there are limited data on its long-term incidence. In our cohort, we recorded a total of 8 episodes of acute pancreatitis (1.4%), with 2 cases (0.3%) experiencing repeated episodes. These numbers are slightly lower than those reported by other authors but are consistent with findings from a study by Kanamori, which included a large group of patients with long-term follow-up.^15^

Kanamori’s study identified several individual predictors of biliopancreatic complications, including sex, endoscopic sphincterotomy, stone size, bile duct diameter, and the coexistence of choledocholithiasis. In our study, we found that bile duct dilation above 8 mm was associated with a higher risk of long-term complications, which is consistent with previous studies.^3^

Diversion of the bile duct to the digestive tract due to the inability to access it through the Vater papilla was also associated with an increased risk of long-term complications (*P* = .002; OR 10.39). However, the number of patients who underwent this novel technique in our study was limited, as it was not widely performed during the study period.

Furthermore, we observed that patients over 50 years of age at the time of ERCP were more likely to develop benign complications. Kanamori’s^16^ study also reported a higher number of complications in patients older than 80 years.^15^ These findings suggest that age may be a significant factor contributing to the development of complications.

Overall, our study provides important insights into the incidence and risk factors associated with long-term complications following ERCP. It underscores the need for careful consideration of patient characteristics and indications for the procedure to minimize the risk of complications, particularly in older patients.

In our study, it was notable that almost half of the patients did not undergo cholecystectomy either before or after ERCP, which may be attributed to their advanced age and associated comorbidities.^17^ Surprisingly, we found that having undergone cholecystectomy (either before or after ERCP) increased the risk of developing long-term benign complications. These findings contrast with what has been reported in other studies,^18^ where post-ERCP cholecystectomy is associated with an overall decrease in biliopancreatic complications following ERCP.

Given this discrepancy, it is crucial to design more prospective studies to clarify the optimal approach to cholecystectomy after ERCP with sphincterotomy in patients with cholelithiasis. Further investigation is needed to determine whether there are specific age groups or patient characteristics that would benefit from post-ERCP cholecystectomy to prevent long-term complications. Such studies would help provide clearer guidelines and recommendations for the management of patients undergoing ERCP in relation to cholecystectomy. 

The development of tumors in the biliopancreatic area following ERCP with sphincterotomy has been a subject of concern and debate. Previous studies have presented conflicting findings regarding the association between sphincterotomy and the development of biliary and pancreatic neoplasms.^10,19-21^

One study by Kalaitzis^10^ in 2012 suggested that endoscopic sphincterotomy, with a mean follow-up of 42 months, may cause the development of biliary epithelial atypia but interpreted it as a reactive-proliferative phenomenon rather than a preneoplastic condition.^22^ However, the association between surgical sphincterotomy and biliary-enteric anastomoses with the development of biliary carcinogenesis has been well documented.^10,22^

The occurrence of biliary carcinogenesis is considered to be a multifactorial process. It is hypothesized that the loss of the sphincter barrier may initiate this process by allowing bacterial colonization, chronic inflammation, and the production of cytotoxic substances.^23^

Several studies have investigated the incidence of tumors in the biliopancreatic area following ERCP with sphincterotomy. A Danish study found a higher incidence of cholangiocarcinoma in the first year after sphincterotomy, but the number of tumors equalized with the control group during long-term follow-up.^19^ Other studies from Finland and Sweden, with a large number of patients who underwent sphincterotomy, did not find significant differences in the long-term incidence of tumors compared to the control group.^22^

In our study, the incidence of biliopancreatic neoplasms aligns with previous reports and oncology registries.^24^ The occurrence of cholangiocarcinoma, pancreatic cancer, intrahepatic hepatocellular carcinoma, and ampulloma in our cohort falls within the expected percentages reported in oncology registries. Therefore, it is challenging to establish a direct cause-and-effect relationship between ERCP with endoscopic sphincterotomy and the subsequent development of biliopancreatic neoplasms.

Further research and long-term prospective studies with the control group are needed to provide more conclusive evidence on the potential association between ERCP with sphincterotomy and the development of biliopancreatic tumors.

The absence of a control group is indeed a limitation, as it would have allowed for a comparison with patients who did not undergo ERCP or sphincterotomy. Including a control group would have provided valuable insights into the specific risks and complications associated with the procedures.

## Figures and Tables

**Table 1.** Patients Characteristics and Follow-Up

		N = 576 (%)
Demographic variables and comorbidity	Age Male sex Female sex HBP DM Hepatopathy Chronic pancreatitis Peptic ulcer IBD No bille duct neoplasms Obesity Smoking Alcoholism	71 (IQR 64-81) 269 (46.5) 307 (53.5) 384 (60.2) 137 (23.7) 36 (6.2) 16 (2.8) 68 (11.8) 5 (0.9) 113 (19.6) 92 (16.0) 103 (23.7) 64 (11.1)
Previous biliary surgery	3	51 (8.8)
Cholecystectomy	Pre-ERCP Post-ERCP	440 (76.4) 197 (34.2) 243 (42.2)
Follow-up time (months)	3	131 (IQR 69-149)
End of follow-up reasons	Death for ERCP complications Death for another reasons Loss of follow-up Study end	5 (0.9) 184 (31.9) 115 (20.0) 272 (47.2)

DM, diabetes mellitus; ERCP, endoscopic retrograde cholangiopancreatography; HBP, high blood pressure; IBD, inflammatory bowel disease; IQR, intercuartile range.

**Table 2.** Variables in Relation to the ERCP Index

	N = 576 (%)
Indication of ERCP Cholestasis AP Choledocholithiasis Cholecystopancreatitis Biliary colic Cholangitis Biliary fistula Hydatid cyst Others ERCP diagnosis: Choledocholithiasis Biliary sludge Benign bile duct stenosis Biliary fistula Normal BD Others Stent placement Cytology Precut	159 (27.6) 122 (21.2) 134 (23.3) 30 (5.2) 28 (4.9) 81 (14.1) 14 (2.4) 2 (0.3) 6 (1%) 262 (45.5) 119 (20.7) 144 (25.0) 10 (1.8) 30 (5.2) 12 (2.1) 97 (16.8) 21 (3.6) 33 (5.7)
Dilated BD	333 (57.6)
Repeat ERCP ERCP complications Others	141 (24.5) 94 (16.3) 47 (8.1)

AP, acute pancreatitis; BD, bile duct; ERCP, endoscopic retrograde cholangiopancreatography.

**Table 3.** Long-Term Benign Post-ERCP Complications

	N = 576 (%)
Overall	94 (16.3)
Choledocholithiasis > 1 episode	68 (11.8) 15 (2.6)
Cholangitis sine materia > 1 episode	26 (4.7) 8 (1.4)
Acute pancreatitis > 1 episode	8 (1.4) 2 (0.3)

**Table 4.** Factors Associated with ERCP Complications

	Overall Complications Univariate	Overall Complications Multivariate, *P*/OR (95% CI)	Benign Complications Univariate, n = 94 (%)	Benign Complications Multivariate, *P*/OR (95% CI)
Age > 50 years	95 (18.5); *P* = .04	NS	89 (17.3); *P* = .04	.039/2.99 (1.05-8.46)
Stent placement	34 (35.1); *P* < .001	*P* < .001/3.01 (1.73-5.25)	32 (33.0); *P* < .001	.0001/2.93 (1.64-5.22)
Dilated BD	72 (21.6); *P* = .002	*P* = .021/1.81 (1.09-2.99)	67 (20.1); *P* = .003	.034/1.77 (1.05-2.99)
Previous biliary surgery	19 (37.3); *P* = .001	*P* = .13/2.36 (1.19-4.65)	19 (37.3); *P* = .001	.006/2.61 (1.31-5.19)
Diversión BD to DT	7 (70.0); *P* < .001	*P* = .002/10.39 (2.3-46.96)	7 (70.0); *P* < .001	.001/12.65 (2.67-59.79)
Cytology	10 (47.6); *P* < .001	*P* = .03/3.14 (1.12-8.19)	11 (52.4); *P* < .001	.003/5.07 (1.76-14.58)
Repeat ERCP	34 (23.8); *P* = .022	NS	34 (23.8); *P* = .006	NS
Biliary fistula	4 (40.0); *P* = .04	NS	4 (40.0); *P* = .038	NS
Cholecystectomy	89 (35.1); *P* < .001	*P* = .001/3.48 (1.68-7.21)	85 (19.3); *P* = .001	.0001/5.17 (2.18-12.2)
Cholecystectomy before ERCP	56 (27.9); *P* = .001	NS	32 (26.2); *P* = .001	NS

BD, bile duct; DT, digestive tract; ERCP, endoscopic retrograde cholangiopancreatography.

**Table 5.** Factors Associated with ERCP Benign Complications

	Choledocholihtiasis Univariate, n = 70 (%)	Choledocholithiasis Multivariate, *P*/OR (95% CI)	Cholangitis “Sine Materia” Univariate, n = 27 (%)	Cholangitis “Sine Materia” Multivariate, *P*/OR (95% CI)	Acute Pancreatitis Univariate, n = 8 (%)	Acute Pancreatitis Multivariate, *P*/OR (95% CI)
Dilated BD	55 (16.5); *P* < .001	.003/2.54 (1.36 - 4.73)	NS	NS	NS	NS
Stent placement	24 (24.7); *P* < .001	.003/2.54 (1.36-4.73)	13 (13.4); *P* < .001	0.001 4,38 (1.87 - 10.25)	NS	NS
Previous biliary surgery	13 (25.5); *P* = .002	.048/2.1 (1.08-4.38)	6 (11.8); *P* = .012	NS	NS	NS
Diversión BD to DT	4 (40.0); *P* = .006	NS	5 (50.0); *P* < .001	0.001 15,93 (3.91 - 64.83)	NS	NS
Cytology	10 (47.6); *P* < .001	.0001/7.85 (2.72-22.68)	5 (23.8); *P* = .002	NS	NS	NS
Repeat ERCP	26 (18.2); *P* = .01	NS	NS	NS	NS	NS
Biliary fistula	NS	NS	2 (20.0); *P* = .017	NS	1 (10.0); *P* = .019	NS
Cholecistectomy	64 (14.5); *P* = .001	.001/5.16 (1.96-13.6)	NS	NS	NS	NS
Cholecistectomy before ERCP	24 (19.7); *P* = .004	NS	NS	NS	NS	NS
Benign BD stenosis	NS	NS	11 (7.5); *P* = .045	NS	NS	NS
Chronic pancreatitis	NS	NS	3 (18.8); *P* = .036	NS	NS	NS

BD, bile duct; DT, digestive tract; ERCP, endoscopic retrograde cholangiopancreatography.
